# Gut Microbiome and Microbiome-Derived Metabolites in Patients with End-Stage Kidney Disease

**DOI:** 10.3390/ijms241411456

**Published:** 2023-07-14

**Authors:** Takeo Koshida, Tomohito Gohda, Takuya Sugimoto, Takashi Asahara, Rin Asao, Isao Ohsawa, Hiromichi Gotoh, Maki Murakoshi, Yusuke Suzuki, Yuichiro Yamashiro

**Affiliations:** 1 Department of Nephrology, Faculty of Medicine, Juntendo University, Bunkyo-ku 113-8421, Tokyo, Japan; 2 Yakult Central Institute, Yakult Honsha Co., Ltd., Kunitachi-shi 186-0012, Tokyo, Japan; 3 Department of Internal Medicine, Saiyu Soka Hospital, Soka-shi 340-0041, Saitama, Japan; 4 Probiotics Research Laboratory, Graduate School of Medicine, Juntendo University, Bunkyo-ku 113-8421, Tokyo, Japan

**Keywords:** diabetic kidney disease, microbiome, uremic toxin

## Abstract

The composition of the gut microbiome is altered in patients with chronic kidney disease (CKD). Dysbiosis leads to decreased levels of stool organic acids (OAs) and systemic inflammation, followed by accumulation of uremic toxins (UTs) and the development of end-stage kidney disease (ESKD). We assessed the relationship between the microbiome and UT levels or the development of ESKD by comparing patients undergoing hemodialysis (HD) and those with normal renal function (NRF). This cross-sectional study recruited 41 patients undergoing HD and 38 sex- and age-matched patients with NRF, and gut microbiome, levels of plasma UTs, inflammatory markers, and stool OAs were compared. The indices of beta-diversity differed significantly between patients with NRF and those undergoing HD, and between patients undergoing HD with and without type 2 diabetes. The levels of stool total OA, inflammatory markers, and UTs differed significantly between the patients with NRF and those undergoing HD. The combined main effects of type 2 diabetes and kidney function status were accumulation of indoxyl sulfate and p-cresyl sulfate. The relative abundances of *Negativicutes* and *Megamonas* were associated with development of ESKD and with the levels of UTs, even after adjustment for factors associated with the progression of ESKD. The present study indicates that the gut environment differs between patients with NRF and those undergoing HD and between patients undergoing HD with and without type 2 diabetes. Moreover, ESKD patients with diabetes accumulate more UTs derived from the gut microbiome, which might be associated with cardio-renal diseases and poor prognosis.

## 1. Introduction

The gut is the largest microecosystem in the human body and plays an important role in human health and diseases [1]. Metabolites produced by the microbiome, such as short-chain fatty acids (SCFAs), uremic toxins (UTs), and lipopolysaccharide (LPS), affect human body homeostasis, and are associated with the development and/or progression of chronic kidney disease (CKD) and/or type 2 diabetes [2,3].

The composition of the gut microbiome has been reported to be profoundly altered in patients with CKD or type 2 diabetes [4,5]. SCFAs are important factors in the gut environment, and dysbiosis induces changes in the SCFA profile. Furthermore, SCFAs have therapeutic potential for the treatment of kidney disease, an effect that is thought to be mediated through the regulation of inflammation via the SCFA receptor G protein-coupled receptor (GPR)-43 and GPR109A [6]. Decreased SCFA production results in accumulation of α-amino nitrogen, which can be transformed into UTs by the gut microbiome [7]. Indoxyl sulfate (IS), p-cresyl sulfate (pCS), and phenyl sulfate (PS) are representative toxins produced by microbiome. IS promotes endothelial dysfunction, leading to the development of cardiovascular and renal problems [8,9]. pCS contributes to renal fibrosis and progression of kidney injury [8,9,10,11]. PS has toxic effect on podocytes, leading to the onset of albuminuria in diabetic kidney disease (DKD) [10]. Microbiome-derived metabolites such as SCFAs and UTs are reportedly related to each other and to inflammation-related factors, including LPS. For example, treatment with propionic acid has been shown to result in decreased serum levels of IS and pCS and increased serum levels of the anti-inflammatory cytokine interleukin (IL)-10 in patients undergoing hemodialysis (HD) [11]. Serum levels of IL-6 have been reported to correlate positively with the serum levels of IS and pCS in patients with CKD [12]. LPS, a major component of Gram-negative bacteria, is thought to be a kidney toxin. Indeed, LPS induces acute kidney injury (AKI) in animal models [13]; however, it remains unclear whether LPS is associated with impairment of renal function in patients with CKD.

Diabetes mellitus is thought to be exacerbated by dysbiosis, leading to decreased SCFA production in the gut [3]. Compared with other kidney diseases, DKD progresses rapidly. In the present study, we hypothesized that differences in the gut microbiome between DKD and non-diabetic CKD are involved in the development of end-stage kidney disease (ESKD). Thus, the gut environment, including the microbiome and the SCFA levels, interact closely with renal dysfunction and accumulation of UTs, but it remains unclear how these factors interact, especially in patients undergoing HD, and whether the gut microbiome differs between patients undergoing HD with and without type 2 diabetes. Therefore, we sought to analyze the gut environment of patients undergoing HD, specifically in comparison with patients with normal renal function (NRF), and to verify our hypothesis that dysbiosis induces dysregulation of stool organic acids (OAs) and results in the elevation of plasma LPS, in turn resulting in a decline in renal function and accumulation of UTs. Notably, this study sought to identify key bacterial subgroups and OA that contribute to the progression to end-stage kidney disease (ESKD).

## 2. Results

### 2.1. Clinical and Biochemical Characteristics

The clinical characteristics of each group are shown in Table 1 and Supplementary Table S1. The patients undergoing HD tended to be older and to exhibit lower body mass indices (BMIs) than those with NRF. On the other hand, systolic blood pressure (BP) and inflammatory markers such as lipopolysaccharide binding protein (LBP) and high-sensitivity C-reactive protein (hsCRP) in patients undergoing HD were significantly higher than those in patients with NRF. The patients with NRF and those undergoing HD showed comparable characteristics with respect to sex, diastolic BP, and type 2 diabetes rates. The patients undergoing HD were more likely to be taking proton pump inhibitor (PPI) medications and phosphorus adsorbents than patients with NRF, but the rates of use of dipeptidyl peptidase-4 (DPP-4) inhibitor, α-glucosidase inhibitor (α-GI), glinide, or glucagon-like peptide-1 receptor agonist (GLP-1RA) in the patients with HD were similar to those in patients with NRF. None of the patients undergoing HD used sodium-glucose cotransporter-2 (SGLT2) inhibitor, metformin, or insulin.

### 2.2. Microbial Diversity

**Figure 1:**
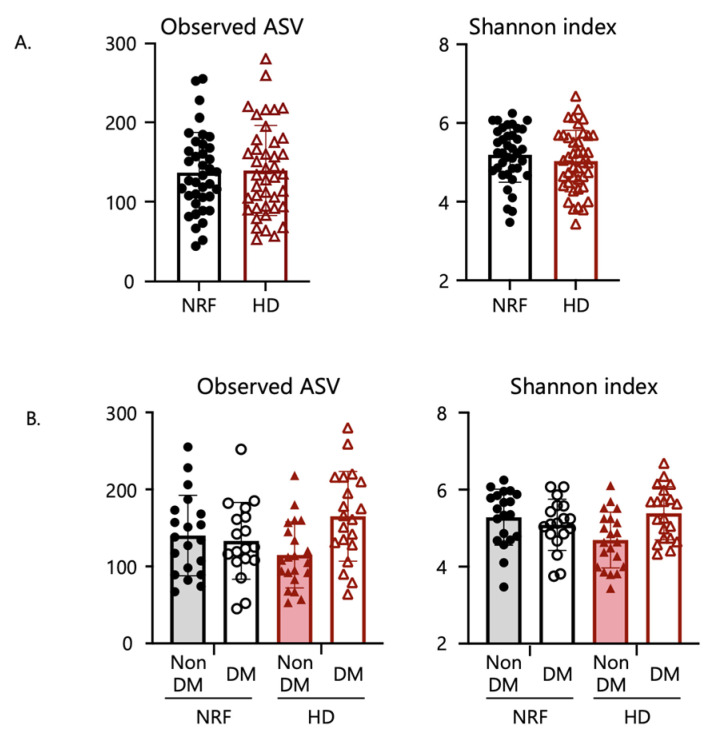

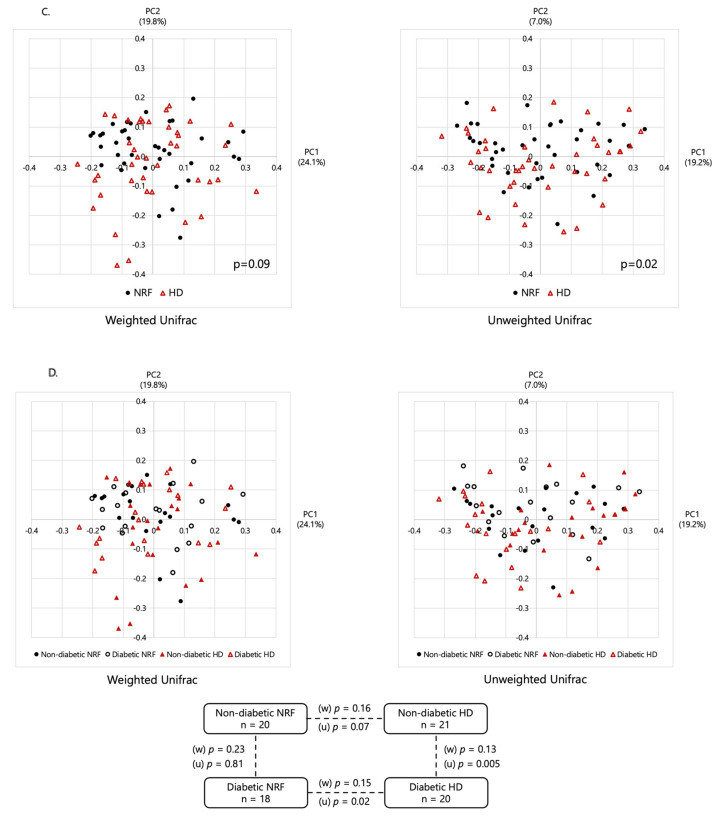
Comparison of gut microbial diversity. Panels (**A**,**B**) show comparisons of α-diversity, including observed ASV and Shannon index, whereas (**C**,**D**) show comparisons of β-diversity and characteristics of the microbial community composition based on weighted (w) and unweighted (u) Unifrac distance by PCoA plots. Panels (**A**,**C**) show comparisons between patients with NRF and those undergoing HD. Panels (**B**,**D**) show comparisons among patients with NRF with and without type 2 diabetes and those undergoing HD with and without type 2 diabetes. The *p*-values in panel (**A**) were calculated by Student’s *t*-test; in panel (**B**) by two-way analysis of variance (ANOVA); and in panels (**C**,**D**) by permutational multivariate analysis of variance (PERMANOVA) based on Unifrac distance.

The α-diversity indices of the microbiome did not differ between patients with NRF and those undergoing HD (Figure 1A) and did not differ depending on kidney function. These indices also did not differ according to diabetic or renal function status (Figure 1B).

As shown in Figure 1C,D, there was a significant difference in β-diversity based on the unweighted Unifrac distance between patients with NRF and those undergoing HD (permutational multivariate analysis of variance (PERMANOVA), *p* = 0.02), and between patients undergoing HD with and without type 2 diabetes (*p* = 0.005). The Jaccard and Bray–Curtis indices are shown in Supplementary Figure S1A,B. These results suggested the existence of structural alterations in the gut microbiome depending on renal function and the underlying etiology of ESKD, i.e., non-diabetes or diabetes. However, β-diversity analysis based on weighted Unifrac distance showed only nominal differences between patients with NRF and those undergoing HD (*p* = 0.09), and between patients undergoing HD with and without type 2 diabetes (*p* = 0.13) (Figure 1C,D). Thus, the fecal microbial structure differed significantly between these groups, with effects apparently depending on kidney function and the cause of ESKD.

### 2.3. Bacterial Taxonomic Differences

**Figure 2:**
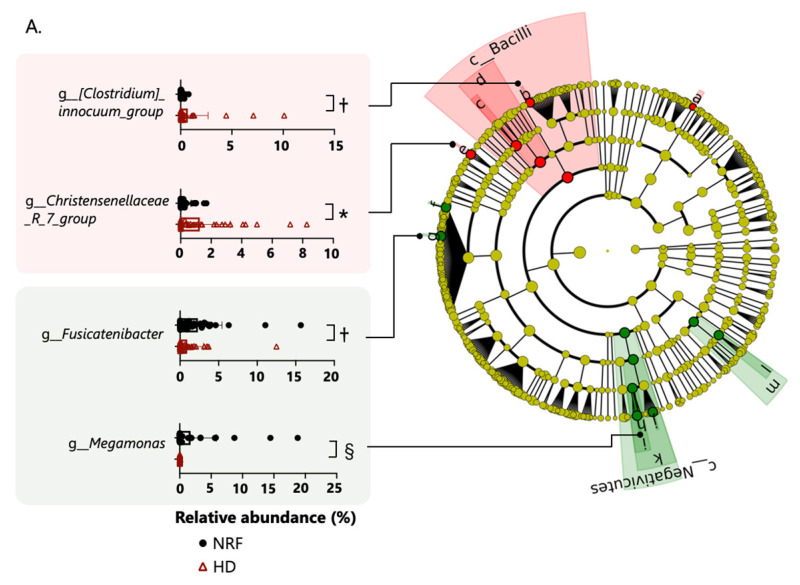

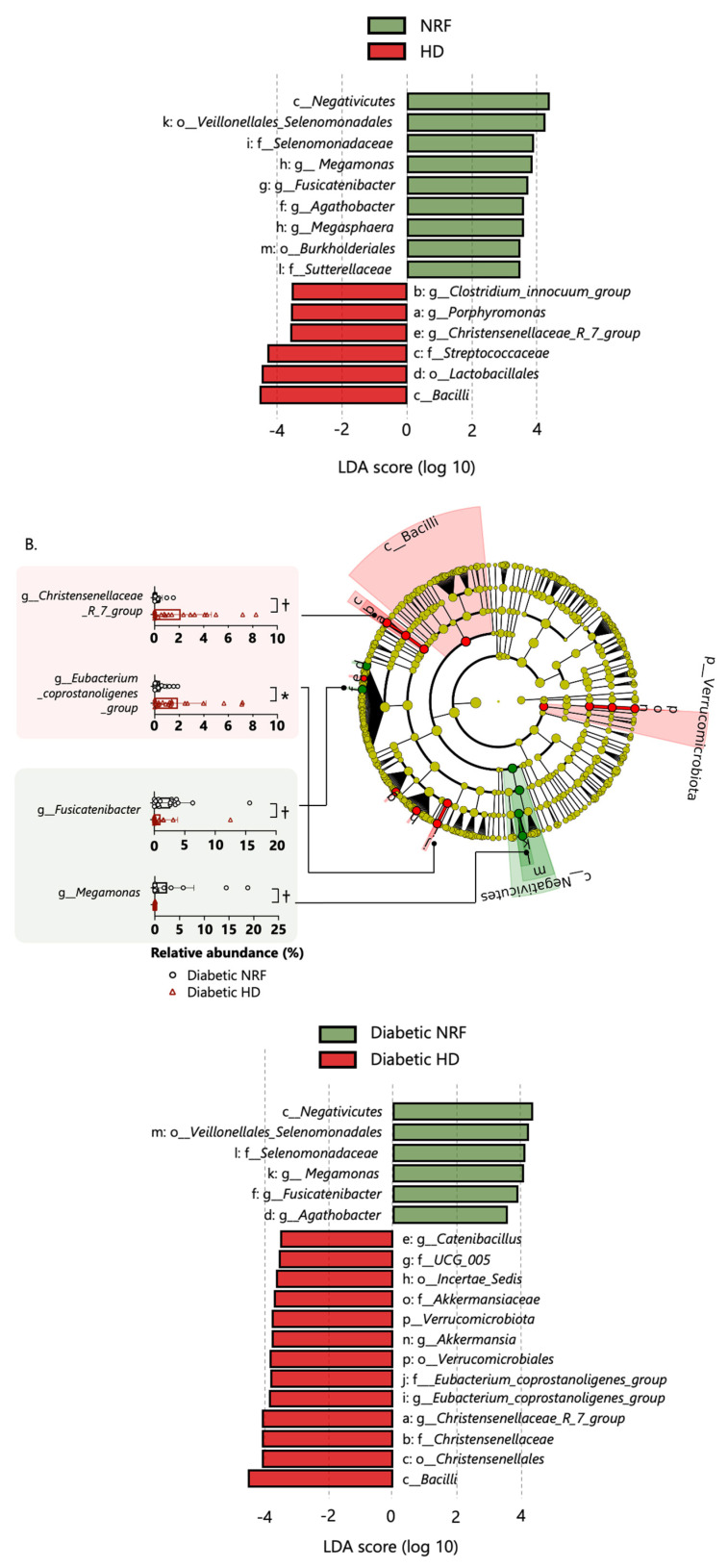

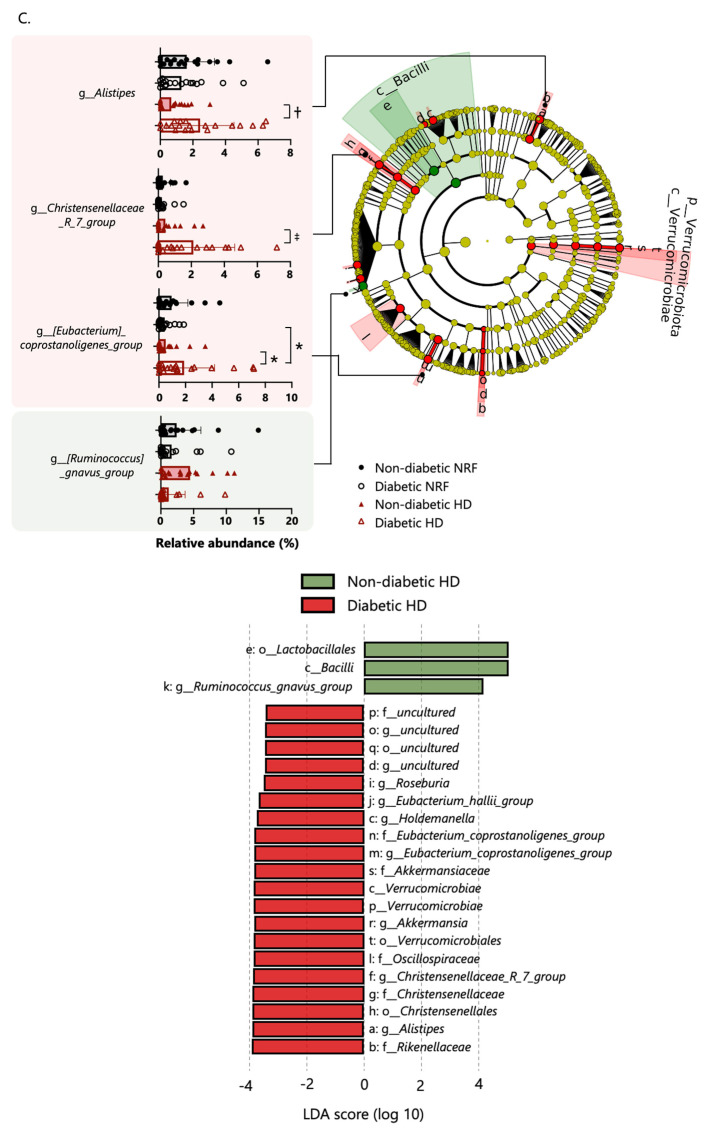
Panel (**A**) shows the enriched bacterial taxa in patients with NRF and those undergoing HD, panel (**B**) shows enriched taxa in diabetic patients with NRF and those undergoing HD, and panel (**C**) shows enriched taxa in patients undergoing HD with and without type 2 diabetes. All comparisons were made using the linear discriminant analysis (LDA) Effect Size method. Only taxa with absolute log(LDA) scores exceeding 3.5 are shown. *: *p* < 0.05, ^†^: *p* < 0.01, ^‡^: *p* < 0.001, ^§^: *p* < 0.0001. The *p*-values in panels (**A**,**B**) are calculated by Student’s *t*-test and in panel (**C**) by two-way ANOVA.

The gut microbiome composition of patients undergoing HD did not differ from that of patients with NRF at the phylum level. Based on linear discriminant analysis effect size (LefSe) analysis, one class, one order, one family, and three genera were attributed to patients undergoing HD; and one class, two orders, three families, and four genera were attributed to patients with NRF (Figure 2A). The following genera demonstrated differential abundance: Christenellaceae_R_7_group (Christensenellaceae family), Clostridium innocuum group (Erysipelotrichaceae family), and other genera were more abundant in patients undergoing HD; and Megamonas, Fusicatenibacter, and other genera were more abundant in patients with NRF. Among patients with type 2 diabetes, one phylum, one class, two orders, three families, and six genera were more abundant in diabetic patients undergoing HD, and one class, one order, one family, and three genera were more abundant in diabetic patients with NRF (Figure 2B). In patients with type 2 diabetes, Christenellaceae_R_7_group (Christensenellaceae family), Megamonas, Fusicatenibacter, and Agathobacter showed similar changes between patients with NRF and those undergoing HD. We further compared the microbial composition between patients undergoing HD with and without type 2 diabetes. Among patients undergoing HD, one phylum, one class, three orders, six families, and nine genera were more abundant in patients undergoing HD with type 2 diabetes, and one class, one order and one genus were more abundant in patients undergoing HD without type 2 diabetes (Figure 2C).

### 2.4. Organic Acids and pH

**Figure 3:**
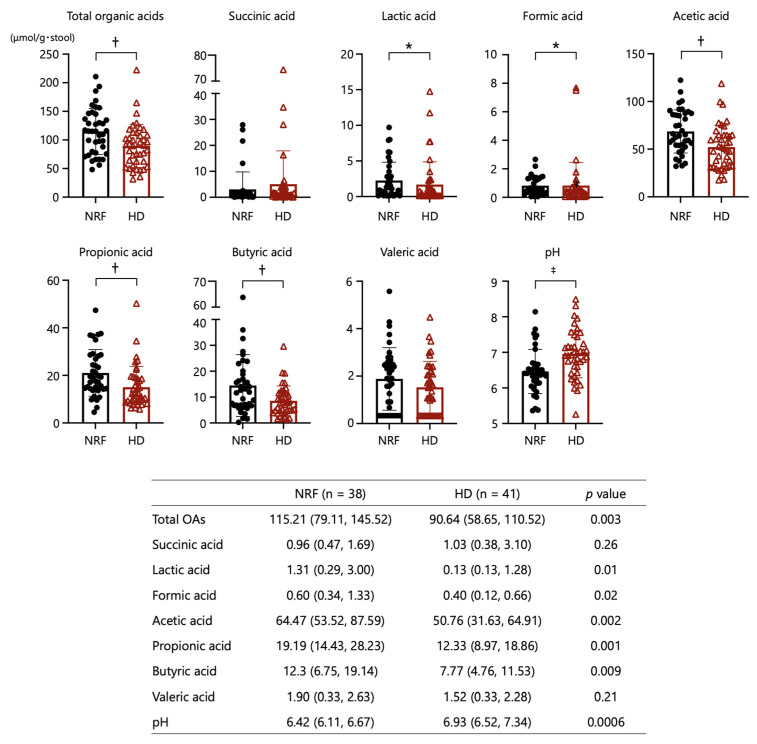
Comparisons of organic acids and pH in stool between patients with NRF and those undergoing HD. *: *p* < 0.05, ^†^: *p* < 0.01, ^‡^: *p* < 0.001. The *p*-values for comparisons between patients with NRF and undergoing HD were calculated by Student’s *t*-test.

As shown in Figure 3, the fecal concentrations of total OAs, acetic acid, propionic acid, butyric acid, lactic acid, and formic acid in the HD group were significantly lower than those in patients with NRF. Fecal pH in patients undergoing HD was significantly higher than that in patients with NRF. The concentrations of the respective OAs were not associated with renal function or diabetes status (Supplementary Figure S2).

### 2.5. Uremic Toxins

**Figure 4:**
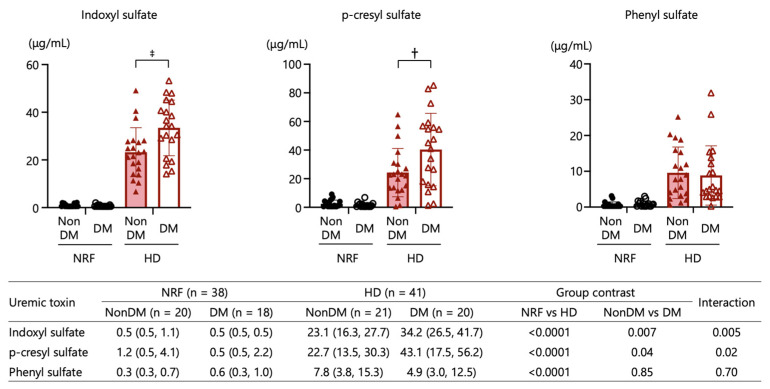
Comparisons of plasma uremic toxins (indoxyl sulfate, p-cresyl sulfate, and phenyl sulfate) among patients with NRF with and without type 2 diabetes and those undergoing HD with and without type 2 diabetes. ^†^: *p* < 0.01, ^‡^: *p* < 0.001. The *p*-values for comparisons between each group were calculated by two-way ANOVA.

All UT levels were significantly higher in patients undergoing HD than in those with NRF. UT levels were significantly associated with renal function status. Also, IS and pCS levels exhibited significant associations with diabetes status. The combined main effects of type 2 diabetes and kidney function status resulted in the highest IS and pCS levels in patients undergoing HD with type 2 diabetes compared with those undergoing HD without type 2 diabetes, giving an interaction by two-way ANOVA *p*-value of 0.005 for IS and *p* = 0.02 for pCS (Figure 4). These results suggest that the uremic state is more severe in patients undergoing HD with type 2 diabetes compared with those without type 2 diabetes.

### 2.6. Functional Prediction

The microbiome function prediction obtained using PICRUSt2 revealed that 10 pathways were upregulated and 9 pathways were downregulated in patients undergoing HD compared with those with NRF (Supplementary Figure S3A). Most of these differentially regulated pathways were related to metabolic functions. Functional categories in patients with NRF, including carbohydrate metabolism (glyoxylate and dicarboxylate; glycolysis/gluconeogenesis; etc.), amino acid metabolism (arginine and proline; histidine, valine, leucine, and isoleucine; etc.), and metabolism of cofactors and vitamins (riboflavin and vitamin B6) showed highly significant differential regulation compared with patients undergoing HD. Pathways showing statistically significantly greater differential regulation between diabetic patients with NRF and undergoing HD (Supplementary Figure S3B), or between patients undergoing HD with and without type 2 diabetes (Supplementary Figure S3C).

### 2.7. Associations among Organic Acids, Inflammatory Markers, and Uremic Toxins

Table 2 shows the correlation coefficient matrix for associations among the levels of OAs, inflammatory markers, and UTs. There was no correlation between the levels of OA and inflammatory markers. On the other hand, a moderate to strong correlation was observed among the levels of acetic acid, propionic acid, and butyric acid. However, the levels of these three OAs were associated only weakly with those of other OAs (formic acid and lactic acid), although a moderate correlation was observed between the levels of formic acid and lactic acid. The OAs could be divided into two categories according to the value of the correlation coefficients. Specifically, a moderate correlation was observed between the levels of hsCRP and LBP. A strong correlation was observed among UTs, and a weak to moderate correlation was observed between each UT and total OAs or LBP. Supplementary Table S2 shows the correlation coefficient among demographical data, clinical parameters, drugs, organic acids, inflammatory markers, uremic toxins, and gut bacteria.

### 2.8. Microbiomes Associated with ESKD or Uremic Toxins

A s shown in Table 3, many of the OAs and inflammatory markers (except for butyric acid, formic acid, and hsCRP) exhibited significant associations with ESKD in simple logistic regression analysis. Logistic analysis was performed to detect relevant bacteria associated with ESKD (Table 4). Selected bacterial subgroups had high log(LDA) scores in a comparison between patients with NRF and those undergoing HD. In Model 1, most of the microbiomes were associated with ESKD after adjustment for age, sex, and diabetes status. Next, because total OAs and LBP are assumed to change in response to dysbiosis [6,13], we selected these, in addition to the clinical characteristics of Model 1, as confounding factors to avoid overfitting in Model 2. Negativicutes, Selenomonadaceae, Megamonas, and other bacterial subgroups were associated with ESKD after adjustment for age, sex, total OA, LBP, and diabetes status. In multiple linear regression analysis adjusted by age, sex, total OAs, and LBP, Megamonas, and the Christensenellaceae_R_7_group (Christensenellaceae family), and other bacterial subgroups showed associations with the levels of IS and pCS (Table 5). Fusicatenibacter was associated only with the level of IS; the NK4A214_group (Oscillospiraceae family), etc., was only associated with the level of pCS; and GCA-900066755 (Lachnospiraceae family), etc., only with the level of PS. Bacterial subgroups that were correlated with the level of UTs were selected in this analysis and are listed in Supplementary Table S3.

## 3. Discussion

Our study provided comprehensive profiling of the gut microbiome and related metabolites, including plasma UTs, in Japanese ESKD patients undergoing HD. Notably, our results provide the first detailed characterization of the gut environment, including not only the microbiome but also metabolites such as OAs, UTs, and LBP, as well as candidate bacterial subgroups for which abundance is associated with ESKD independent of other factors associated with ESKD. Furthermore, our data demonstrated that the gut microbiome differs remarkably between patients undergoing HD with and without diabetes. These insights indicate the possibility that a different etiology involving the gut environment may exist between ESKD patients with diabetes and without. Unique gut environment may contribute to the poorer prognosis of ESKD patients with diabetes.

SCFAs are a source of nutrients for intestinal epithelial cells and have been shown to facilitate the maintenance of intestinal barrier function [2]. Dysbiosis contributes to intestinal barrier dysfunction and systemic inflammation through decreases in the fecal levels of SCFAs [13]. On the other hand, supplementation of SCFAs is known to ameliorate renal inflammation in multiple kidney diseases. In mice with AKI induced by ischemia and reperfusion, SCFA supplementation ameliorates hypoxia in kidney epithelial cells by improving mitochondrial biogenesis [13]. SCFA-treated mice with diabetes are protected from nephropathy through decreased expression of inflammatory cytokines, chemokines, and fibrosis-promoting proteins, but this effect is not seen in mice lacking GPR43 or GPR109A metabolite-sensing receptors. In vitro, SCFAs ameliorate inflammation in renal tubular cells and podocytes under hyperglycemic conditions [6]. Acetic acid inhibits LPS-induced secretion of tumor necrosis factor (TNF)α by murine and human mononuclear cells, an effect that is mediated by activation of GPR43 [14]. Butyric and propionic acids reduce the expression of TNFα and nitric oxide synthase (NOS) in LPS-induced monocytes [15]. These insights indicate strong relationship between inflammation and the levels of SCFAs. However, in the present study, the levels of fecal SCFAs known to affect dysbiosis did not demonstrate any correlation with inflammatory markers such as hsCRP and LBP.

The fecal levels of OAs, especially SCFAs, have been reported to be significantly decreased in patients with type 2 diabetes and CKD [2,3]. In the present study, the total amount of OAs in stool was associated with renal function, but not with the presence or absence of type 2 diabetes. The accumulation of urea in intra- and extracellular fluids is known to follow declines in renal function, leading in turn to a massive influx of this compound into the gastrointestinal tract. Hydrolysis of urea by urease, which is expressed in some microbial species in the gut microbiome, results in the formation of large quantities of ammonia, leading to elevation of gut pH [4]. Elevation of gut pH has been reported to impede production of butyrate [16], and drastic changes in the intestinal environment may influence the production of SCFAs. In addition, restriction of dietary fiber such as fruits and vegetables in patients undergoing HD may contribute, in part, to the depletion of SCFAs in stool. In the present study, the relative abundance of *Megamonas*, which has been shown to produce acetic and propionic acid from glucose in vitro [17], was decreased more in patients undergoing HD than those with NRF. Depletion of *Megamonas* in patients undergoing HD is consistent with past reports in patients with CKD [18]. Indeed, the present study detected a weak but significant correlation between the relative abundance of *Megamonas* and the level of acetic acid (*ρ* = 0.25, *p* = 0.03) or propionic acid (*ρ* = 0.25, *p* = 0.03), indicating that the depletion of *Megamonas* may contribute to the aggravation of intestinal inflammation. Changes in the gut microbial composition result in chronic inflammation and metabolic dysfunction, as has been reviewed elsewhere [19]. Decreased lactic and formic acid levels may be related to declines in renal function via distinct mechanisms involving other SCFAs as we did not detect any significant correlation between the level of formic acid and other SCFAs, although a moderate correlation was observed between the levels of lactic acid and formic acid.

Given that UTs are produced in the gut by microorganisms, the levels of UTs are closely associated with the gut microbiome. A scheme depicting the proposed pathway of UT production is shown in Supplementary Figure S4. Li et al. previously demonstrated that higher levels of IS predict all-cause and cardiovascular mortality in patients undergoing HD [20]. In the present study, we showed that the levels of IS, pCS and PS in patients undergoing HD were significantly higher than those in patients with NRF, and there was a strong correlation among UTs. The correlation between UTs derived from different precursors (IS (tryptophan) vs. pCS (tyrosine), IS vs. PS (tyrosine)) was stronger than that between pCS and PS derived from the same precursor (tyrosine). Multiple regression analysis showed that some bacterial subgroups were correlated with both IS and pCS, but those groups correlated with PS were not correlated with IS and pCS. Similar to our study, Itoh et al. showed that the correlation between pCS and PS was not as strong as that between IS and pCS or PS [21]. These results are at least not due to differences in precursors but may be due in no small part to changes in the gut microbiome. Also, the levels of IS and pCS in patients undergoing HD with type 2 diabetes were significantly higher than those in patients undergoing HD without type 2 diabetes, despite similar degrees of renal dysfunction. These results suggest that structural differences in the gut microbiome may influence, in part, the accumulation of UTs. IS is produced from indole, which is derived in turn from tryptophan. pCS and PS are produced from cresol and phenol, respectively, both of which are derived in turn from tyrosine. In silico analysis has demonstrated the presence of indole and cresol biosynthetic pathways in bacteria of the genus *Alistipes* [22]. Interestingly, in the present study, the genus *Alistipes* was enriched in patients undergoing HD with type 2 diabetes compared with those undergoing HD without type 2 diabetes. The increased levels of pCS, but not of PS, in patients undergoing HD with type 2 diabetes may reflect differences in the abundance in the microbiome of cresol- or phenol-producing bacteria. Notably, in vitro culturing has shown that phenol is produced primarily by aerobic gut bacteria, whereas p-cresol is produced primarily by anaerobic gut bacteria [23]. We hypothesize that patients undergoing HD with type 2 diabetes may have an impaired gut environment (as demonstrated by elevation of the levels of IS and pCS) compared with that of patients undergoing HD without type 2 diabetes. This difference may lead to the poorer cardio-renal prognosis seen in patients with CKD who also have type 2 diabetes (compared with those with CKD who do not have type 2 diabetes).

Predicted functional analysis revealed that dysregulation in a taurine-related pathway is associated with a decline in renal function. Taurine exhibits anti-oxidant effects via its uptake in the intestine [24] and also is incorporated as a bile acid conjugate. Bile acids are known metabolites of the intestinal microbiome and may contribute to human health, either directly or via effects on the microbiome. Changes in a taurine-related pathway may indicate a novel etiology linking dysbiosis and CKD progression.

The present study has several limitations. Firstly, the patients with NRF were not entirely healthy, given that these individuals were presenting at hospital (and so were enrolled in the present study) with some chronic lifestyle-related diseases such as type 2 diabetes, hypertension, and dyslipidemia. In the present study, α-diversity indices in patients undergoing HD did not differ from those in patients with NRF, in contrast to the case in a previous study [25]. Several studies have reported that patients with hypertension or type 2 diabetes have lower α-microbial diversity compared with those without such conditions, indicating that type 2 diabetes and hypertension may be strong determinants of α-diversity indices compared with renal function decline [26,27]. In the present study, β-diversity in diabetic patients undergoing HD differed from that in diabetic patients with NRF; in contrast, a previous study indicated that β-diversity did not differ between patients with early- (stage 1 to 3a) and late- (stage 3b to 5) stage DKD [28]. The discrepancy between these two studies may reflect, in part, differences in the severity of DKD between the patients in the two studies. Secondly, the number of study patients was not sufficient to reveal a possible role for the microbiome in ESKD. Thirdly, study patients were solely Japanese individuals, meaning that our results may not necessarily be applicable to patients of other ethnicities. Fourthly, we cannot exclude the influence of therapeutic agents. The usage rates of PPIs and phosphate binders have been reported to affect the gut microbiome [29,30], though these rates were comparable in the present study when comparing patients undergoing HD with or without type 2 diabetes. Medications used to treat type 2 diabetes (e.g., SGLT-2 inhibitors, DPP-4 inhibitors, and α-GI) also have been reported to alter the gut microbiome [31,32,33]. Given the small number of patients taking the α-GI (n = 3; 15% of diabetic HD group) or GLP-1 RA (n = 2; 10% of diabetic HD group), the impact of these drugs on our results was expected to be negligible. On the other hand, 50% of patients undergoing HD with type 2 diabetes were being treated with DPP-4 inhibitors, but the structure of the microbiome did not differ detectably between those individuals who did or did not use DPP-4 inhibitors (β-diversity based on weighted Unifrac distance, *p* = 0.93; unweighted, *p* = 0.56). Although these limitations should be taken into consideration, our findings have the potential to provide new insights into the distinct pathophysiological mechanisms occurring in patients undergoing HD with and without type 2 diabetes.

In conclusion, the gut microbiome and derived metabolites in patients undergoing HD differed from those of patients with NRF. Microbiome analyses identified several bacterial subgroups that may be involved in the progression of renal dysfunction and accumulation of UTs. These differences in the gut microbiome may contribute to pathophysiological mechanisms differing between patients undergoing HD with and without diabetes.

## 4. Materials and Methods

### 4.1. Research Design

The present study enrolled 41 patients undergoing HD who visited the Saiyu Soka Hospital (Saitama, Japan) or Chiba Tokushukai Hospital (Chiba, Japan), as well as 39 patients who regularly visited the Outpatient Clinic of Juntendo University Hospital (Tokyo, Japan) for the management of hypertension, dyslipidemia, or type 2 diabetes without impaired renal function (defined as an estimated glomerular filtration rate (eGFR) of less than 60 mL/min/1.73 m^2^ for 3 months) between January 2019 and October 2020. To compare the gut microbiome and microbiome-derived metabolites between patients with and without diabetes, approximately the same number of diabetic and non-diabetic patients with normal renal function and undergoing HD, respectively, were recruited in the present study. Patients were excluded if they exhibited malignancy, acute intercurrent infections, and/or had been administered antibiotics, probiotics, immunosuppressive drugs, lubiprostone, or erobixibat within one month prior to enrollment in the study. Patients with a history of inflammatory bowel disease or bowel resection also were excluded.

### 4.2. Collection of Stool Samples and DNA Extraction

Fecal samples were placed directly into two tubes (approximately 0.5 g/tube) by the subjects; one tube contained 2 mL of RNAlater^®^ (Thermo Fisher Scientific, Waltham, MA, USA) and zirconia beads, and the other was empty. The tubes subsequently were triple-wrapped and transported to the hospital. On receipt, the samples with RNAlater^®^ were stored at 4 °C (for analysis of the microbiome) and the others were placed in a freezer at −80 °C (for analysis of fecal OA concentration and pH). The patient’s identity and clinical information were unknown to the technician performing the following analyses. Stored fecal samples were pretreated for DNA extraction as described previously [34]. Bacterial genomic DNA was extracted from the resulting samples using the phenol-chloroform method [35].

### 4.3. 16S ribosomal RNA Gene Amplicon Sequencing and Analysis

16S rRNA gene region sequencing was carried out as previously described, with slight modifications [36]. Briefly, the V3–4 regions of the 16S rRNA gene in each sample were amplified using forward (515F) and reverse (806R) primers [37] and an ABI PRISM 7500 Real-Time PCR System (Applied Biosystems, Framingham, MA, USA). The library was constructed by mixing equal amounts of amplicon for every sample. Sequencing was performed using a MiSeq platform with MiSeq Reagent Kits v2 (Cat. No. MS-102-2003; Illumina, San Diego, CA, USA). The amplicon sequence reads were processed using QIIME 2 software (ver. 2020.11). The resulting feature table was used for taxonomic assignment based on the SILVA database (version 138) (https://www.arb-silva.de/ (accessed on 18 January 2021)). α-diversities were represented as the number of observed amplicon sequence variants (ASVs), including estimates of the Shannon index. β-diversity was assessed by weighted and unweighted Unifrac distance.

### 4.4. Measurements of Fecal Organic Acids and pH

The concentrations of OAs in feces were determined using previously described methods [5]. Briefly, a portion of the feces was homogenized in four volumes of 0.15 mol/L perchloric acid and allowed to stand at 4 °C for 12 h. The suspension was centrifuged, and then the filtered supernatant was analyzed using a high-performance liquid chromatography system with a 432 Conductivity Detector (Waters Co., Milford, MA, USA) equipped with two columns (Shodex RS pack KC-811; Showa Denko Co., Ltd., Tokyo, Japan). Fecal pH was measured directly by inserting the glass electrode of a D-51 pH meter (Horiba Seisakusho, Tokyo, Japan) into a sample of homogenized feces.

### 4.5. Measurements of Inflammatory Markers and Uremic Toxins

Enzyme-linked immunosorbent assay (ELISA) was used to measure the level of plasma lipopolysaccharide binding protein (LBP, cat no. HK314-02; Hycult Biotech, Uden, The Netherlands). High-sensitivity C-reactive protein (hsCRP) was measured using nephelometry (Nephelometer II; Dade Behring, Deerfield, IL, USA). The levels of plasma IS, pCS, and PS were measured by LSI Medience Co. (Tokyo, Japan) using liquid chromatography coupled to dual mass spectrometry (LC-MS/MS), as previously described [9].

### 4.6. Functional Prediction of Microbial Communities

The DNA sequence data were analyzed using PICRUSt2 (Phylogenetic Investigation of Communities by Reconstruction of Unobserved States 2), which is a computational approach that predicts the metagenome functional content based on microbial community profiles obtained from 16S rRNA gene sequences [38]. Significant differences in Kyoto Encyclopedia of Genes and Genomes (KEGG) pathways were evaluated by STAMP software.

### 4.7. Statistical Analysis

Statistical analysis was performed in JMP Pro (v. 16.0.0; SAS, Cary, NC, USA) or Prism (v. 9.2.0; GraphPad, San Diego, CA, USA). Continuous variables with and without a normal distribution are presented as mean ± standard deviation or median and interquartile range, respectively. Comparisons between patients with NRF and those undergoing HD were performed by Student’s *t*-test for normal continuous variables and Wilcoxon rank-sum test for non-normal continuous variables. Data with two independent variables were analyzed by two-way ANOVA. Comparison by PERMANOVA based on Unifrac distance was performed to assess whether the microbial communities of the groups differed significantly. The LEfSe method (http://huttenhower.sph.harvard.edu/lefse/) was applied to analyze the characteristics of the fecal microbiome in each group [39]; the effect size of each feature was evaluated via linear discriminant analysis (LDA), with a log(LDA) score of 3.5 (above 3.5 or low below −3.5) used as the cut-off value. We assessed the potential relationship between pairs of variables using Spearman’s correlation analysis. Simple and multiple logistic regression analysis were used to evaluate the independence of factors that showed significant differences between patients with NRF and those undergoing HD. Multivariable linear regression analysis was performed to identify factors predicting the levels of UTs.

## Figures and Tables

**Table 1:** Clinical characteristics and inflammatory markers in study patients.

	Normal Renal Function (n = 38)	Hemodialysis (n = 41)	*p* Value
Clinical characteristics			
eGFR (mL/min/1.73 m^2^)	77.1 ± 15.3	NA	
Age (years)	69.1 ± 5.8	71.2 ± 5.0	0.08
Male (%)	65.8%	58.5%	0.64
BMI	24.3 ± 3.2	22.8 ± 3.7	0.07
Systolic BP (mmHg)	127 ± 12	149 ± 24	<0.001
Diastolic BP (mmHg)	75 ± 9	77 ± 15	0.61
Type 2 diabetes (%)	47.4%	48.8%	>0.999
Inflammatory markers			
hsCRP (mg/dL)	0.058 (0.027, 0.113)	0.096 (0.032, 0.219)	0.049
LBP (ng/mL)	13.1 (11.3, 14.4)	15.4 (13.4, 20.0)	<0.001
Medication			
Proton pump inhibitor	7.9%	63.4%	<0.001
Phosphate binder	0.0%	80.5%	<0.001
DPP-4 inhibitor	36.8%	24.4%	0.33
α-GI	5.3%	7.3%	>0.999
Glinide	7.9%	7.3%	>0.999
GLP-1 receptor agonist	0.0%	4.9%	0.49
SGLT2 inhibitor	10.5%	0.0%	0.049
Metformin	15.8%	0.0%	0.01
Insulin	5.3%	0.0%	0.23

Data are shown as the mean ± SD or the median (first quartile, third quartile). BMI, body mass index; BP, blood pressure; DPP-4, dipeptidyl peptidase-4; eGFR, estimated glomerular filtration rate; GI, glucosidase inhibitor; GLP-1, glucagon-like peptide-1; hsCRP, high sensitivity C-reactive protein; LBP, lipopolysaccharide-binding protein; SD, standard deviation; SGLT2, sodium-glucose cotransporter-2; NA, not applicable. *p* values compared between NRF and HD group are calculated by Student’s *t*-test.

**Table 2:** Spearman correlation coefficients among organic acids, inflammatory markers, and uremic toxins.

	Acetic Acid	Propionic Acid	Butyric Acid	Formic Acid	Lactic Acid	hsCRP	LBP	IS	pCS	PS
Total organic acid	0.91 ^§^	0.79 ^§^	0.73 ^§^	0.11	0.47 ^§^	−0.04	−0.21	−0.34 ^†^	−0.33 ^†^	−0.27 *
Acetic acid		0.69 ^§^	0.66 ^§^	0.13	0.37 ^‡^	−0.01	−0.17	−0.34 ^†^	−0.32 ^†^	−0.25 *
Propionic acid			0.57 ^§^	−0.04	0.34 ^†^	0.01	−0.18	−0.28 *	−0.35 ^†^	−0.15
Butyric acid				0.01	0.26 *	0.04	−0.14	−0.26 *	−0.15	−0.19
Formic acid					0.42 ^‡^	0.04	0.00	−0.22	−0.29 *	−0.18
Lactic acid						−0.01	−0.07	−0.30 ^†^	−0.42 ^‡^	−0.26 *
hsCRP							0.67 ^§^	−0.21	0.13	0.24 *
LBP								0.43 ^§^	0.27 *	0.44 ^§^
IS									0.82 ^§^	0.80 ^§^
p-CS										0.60 ^§^

Correlations among organic acids, inflammation markers, and uremic toxins were assessed by Spearman’s rank correlation coefficient analysis. IS, indoxyl sulfate; pCS, p-cresyl sulfate; PS, phenyl sulfate. Other abbreviations are the same as those in Table 1. ^§^ *p* < 0.0001, ^‡^ *p* < 0.001, ^†^ *p* < 0.01, * *p* < 0.05.

**Table 3:** Univariate logistic regression analysis of the organic acids and inflammatory markers for ESKD.

Variable	OR (95% CI)	*p* Value
Total organic acid	0.46 (0.27–0.78)	0.004
Acetic acid	0.43 (0.25–0.74)	0.002
Propionic acid	0.49 (0.29–0.81)	0.005
Butyric acid	0.61 (0.37–1.01)	0.055
Formic acid	0.62 (0.38–1.00)	0.051
Lactic acid	0.54 (0.33–0.87)	0.01
hsCRP	1.63 (0.99–2.71)	0.057
LBP	3.70 (1.70–8.07)	0.001

CI, confidence interval. ESKD, end-stage kidney disease. OR, odds ratio. Other abbreviations are the same as those in Table 1.

**Table 4:** Multivariate logistic regression analysis of the microbiomes for ESKD.

Microbiome	Model 1	Model 2
	OR (95% CI)	
c__Negativicutes	0.37 (0.20, 0.69) ^†^	0.40 (0.20, 0.80) ^†^
o__Veillonellales_Selenomonadales	0.54 (0.33, 0.88) *	0.56 (0.32, 0.98) *
o__Burkholderiales	0.41 (0.22, 0.76) ^†^	0.58 (0.28, 1.22)
f__Selenomonadaceae	0.32 (0.15, 0.68) ^†^	0.30 (0.11, 0.80) *
f__Sutterellaceae	0.39 (0.22, 0.68) ^†^	0.48 (0.24, 0.94) *
g__Agathobacter	0.60 (0.38, 0.95) *	0.74 (0.41, 1.34)
g__Fusicatnibacter	0.55 (0.34, 0.89) *	0.46 (0.25, 0.86) *
g__Megamonas	0.24 (0.08, 0.71) ^†^	0.26 (0.08, 0.86) *
c__Bacilli	2.01 (1.21, 3.35) ^†^	2.25 (1.18, 4.30) *
o__Lactobacillales	1.88 (1.15, 3.07) *	1.38 (0.75, 2.52)
f__Streptococcaceae	1.62 (1.01, 2.60) *	1.42 (0.82, 2.46)
g__Clostridium_innocuum_group ^(^*^1)^	2.14 (1.29, 3.56) *	2.05 (1.04, 4.05) *
g__Christensenellaceae_R_7_group ^(^*^2)^	1.49 (0.94, 2.34)	2.01 (1.06–3.80) *

“c”, “o”, “f”, and “g” indicate class, order, family, and genus (respectively). Other abbreviations are the same as those used in the preceding supplementary tables. Model 1: individual microbiome only. Model 2: Model 1 + age, sex, total organic acid, LBP, and diabetes status. ^†^ *p* < 0.01, * *p* < 0.05. No symbol: no significant change. ^(^*^1)^: Erysipelotrichaceae family, ^(^*^2)^: Christensenellaceae family.

**Table 5:** Multivariate linear regression model for level of each uremic toxin according to the candidate microbiome components.

Microbiome	Model 1: IS	Model 2: pCS	Model 3: PS
	Standardized Coefficient		
o__Burkholderiales	−0.08	−0.13	−0.14
f__Sutterellaceae	−0.13	−0.11	−0.26 *
g__UBA1819 ^(^*^1)^	0.06	0.11	0.16
o__Clostridia.sp	0.23 *	0.41 ^‡^	−0.03
o__Christensenellales	0.26 *	0.38 ^‡^	0.04
o__Clostridia_vadinBB60_group ^(^*^2)^	0.23 *	0.36 ^†^	−0.004
f__Eggerthellaceae	0.23 *	0.24 *	0.10
c__Clostridia;__;__;__	0.23 *	0.41 ^‡^	−0.03
f__Christensenellaceae	0.26 *	0.38 ^†^	0.04
f__Clostridia_vadinBB60_group ^(^*^3)^	0.23 *	0.36 ^†^	−0.004
g__[Clostridium]_innocuum_group ^(^*^4)^	0.14	0.18	0.24 *
c__Clostridia;__;__	0.23 *	0.41 ^‡^	−0.03
g__Christensenellaceae_R_7_group ^(^*^5)^	0.27 ^†^	0.39 ^‡^	0.06
g__Clostridia_vadinBB60_group ^(^*^6)^	0.23 *	0.35 ^†^	−0.004
g__Family_XIII_AD3011_group ^(^*^7)^	0.19	0.34 ^†^	0.06
g__Megamonas	−0.26 *	−0.23 *	−0.13
c__Negativicutes	−0.16	−0.15	−0.08
f__Selenomonadaceae	−0.26 ^†^	−0.23	−0.14
g__Fusicatenibacter	−0.23 *	−0.20	−0.16
o__Clostridia	0.13	0.34 ^†^	0.04
f__Hungateiclostridiaceae	0.13	0.34 ^†^	0.07
f__Oscillospiraceae	0.06	0.18	−0.05
f__Anaerovoracaceae	0.17	0.30 ^†^	0.07
g__Ruminiclostridium	0.13	0.33 ^†^	0.06
g__Intestinimonas	0.22	0.37 ^†^	0.19
g__NK4A214_group ^(^*^8)^	0.20	0.49 ^‡^	−0.03
f__Ruminococcaceae;__	0.10	0.30 ^†^	−0.04
g__Anaerofilum	0.17	0.30 ^†^	0.04
g__Negativibacillus	0.15	0.31 ^†^	−0.01
g__[Eubacterium]_brachy_group ^(^*^7)^	0.09	0.29 ^†^	0.03
o__Clostridiales	0.10	−0.02	0.26 *
f__Clostridiaceae	0.10	−0.02	0.26 *
g__Clostridium_sensu_stricto_1	0.09	−0.03	0.28 *
g__GCA-900066755 ^(^*^9)^	0.12	0.02	0.39 ^‡^
g__Parasutterella	−0.08	−0.07	−0.21

IS: indoxyl sulfate, pCS: p-cresyl sulfate, PS: phenyl sulfate. Other abbreviations are the same as those used in the preceding supplementary tables. Models 1–3: age, sex, total organic acid, LBP, diabetes status, and bacterial group. ^‡^ *p* < 0.001, ^†^ *p* < 0.01, * *p* < 0.05. ^(^*^1)^: Ruminococcaceae family, ^(^*^2)^: Clostridia class, ^(^*^3)^: Clostridia class Clostridia_vadinBB60_group order, ^(^*^4)^: Erysipelotrichaceae family, ^(^*^5)^: Christensenellaceae family, ^(^*^6)^: Clostridia class Clostridia_vadinBB60_group order Clostridia_vadinBB60_group family, ^(^*^7)^: Anaerovoracaceae family, ^(^*^8)^: Oscillospiraceae family, ^(^*^9)^: Lachnospiraceae family.

## Data Availability

The datasets used and/or analyzed during the current study are available from the corresponding author on reasonable request. 16S rRNA gene sequencing data in this paper are available from DDBJ Sequence Read Archive (DRA) under accession number DRA015731.

## Supplementary Materials

The supporting information can be downloaded at: https://www.mdpi.com/article/10.3390/ijms241411456/s1.

## Abbreviations

AKI	acute kidney injury
ANOVA	analysis of variance
ASVs	amplicon sequence variants
BMI	body mass index
BP	blood pressure
CKD	chronic kidney disease
DKD	diabetic kidney disease
DPP-4	dipeptidyl peptidase-4
eGFR	estimated glomerular filtration rate
ELISA	enzyme-linked immunosorbent assay
ESKD	end-stage kidney disease
GLP-1RA	glucagon-like peptide-1 receptor agonist
GPR	G protein-coupled receptor
HD	hemodialysis
hsCRP	high-sensitive c-reactive protein
IL	interleukin
IS	indoxyl sulfate
KEGG	Kyoto Encyclopedia of Genes and Genomes
LBP	lipopolysaccharide-binding protein
LC-MS/MS	liquid chromatography coupled to dual mass spectrometry
LDA	linear discriminant analysis
LEfSe	linear discriminant analysis effect size
LPS	lipopolysaccharide
NOS	nitric oxide synthase
NRF	normal renal function
OA	organic acid
UT	uremic toxin
pCS	p-cresyl sulfate
PERMANOVA	permutational multivariate analysis of variance
PICRUSt2	phylogenetic investigation of communities by reconstruction of unobserved states 2
PPI	proton pump inhibitor
PS	phenyl sulfate
SCFA	short-chain fatty acid
SGLT2	sodium-glucose cotransporter-2
TNF	tumor necrosis factor
α-GI	alpha-glucosidase inhibitor
