# Supplementary material for: Gut Microbiome and Microbiome-Derived Metabolites in Patients with End-Stage Kidney Disease
Source: Int J Mol Sci. 2023 Jul 14;24(14):11456. doi: 10.3390/ijms241411456 (PMC10380578; doi:10.3390/ijms241411456)
Supplement: Supplementary file 1 [file ijms-24-11456-s001.zip › Supplementary Figures.pdf]

Supplementary Figure legends:

Supplementary figure S1A

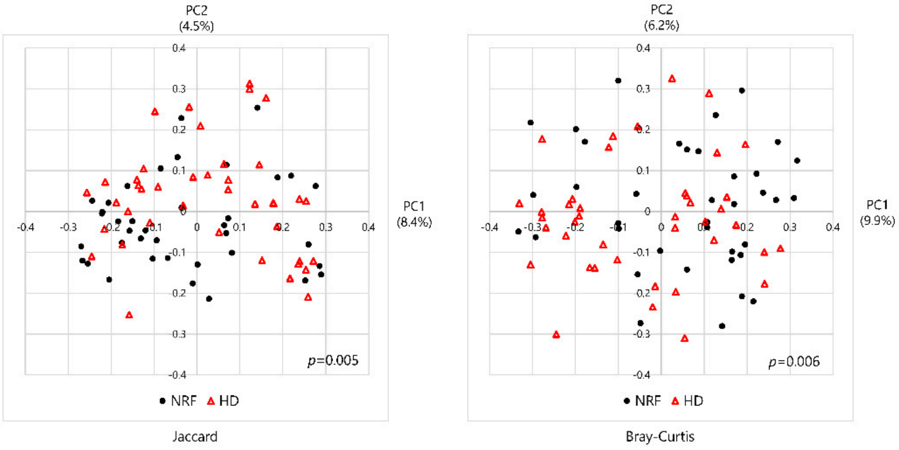

Supplementary figure S1B

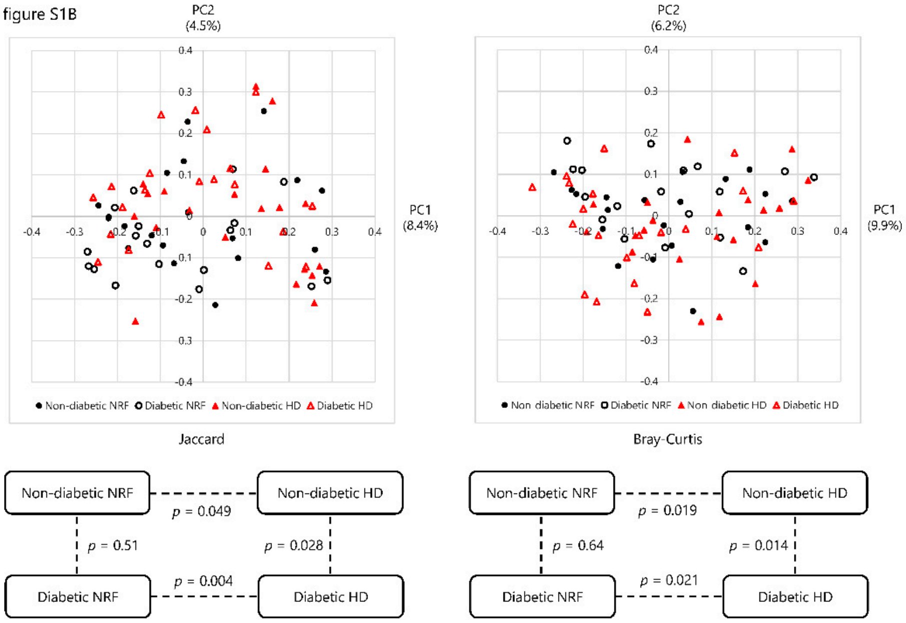

Supplementary Figure S1. Comparison of gut microbial diversity based on Jaccard and Bray-Curtis β-diversity by PCoA plot. Panel A shows the comparison between the NRF and HD groups, and Panel B shows the comparisons among patients with NRF with and without type 2 diabetes and those undergoing HD with and without type 2 diabetes. The *p*-values in panel A and B were calculated by PERMANOVA based on each metric.

Supplementary figure S2

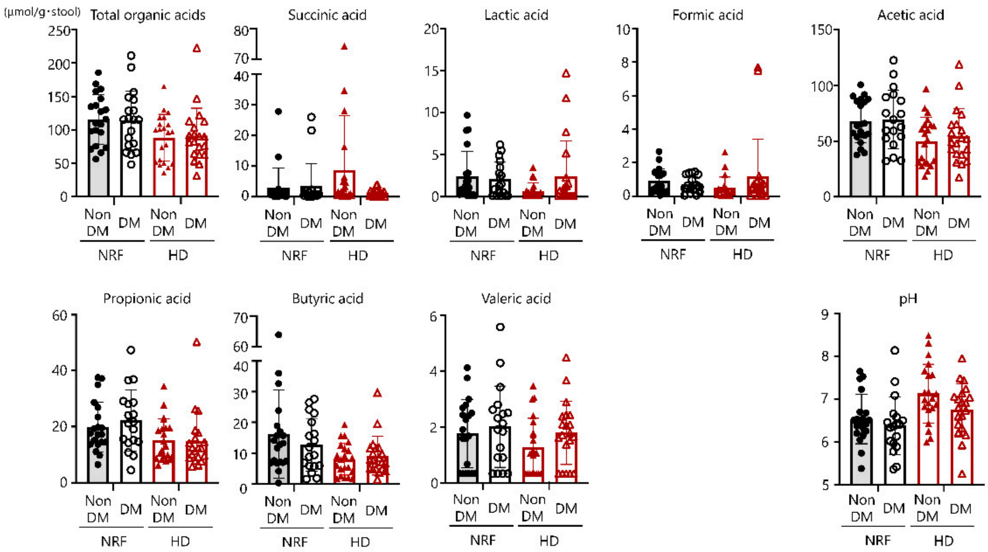

Supplementary Figure S2. Comparisons of organic acids and pH in stool among patients with NRF with and without type 2 diabetes and those undergoing HD with and without type 2 diabetes.

Supplementary figure S3A

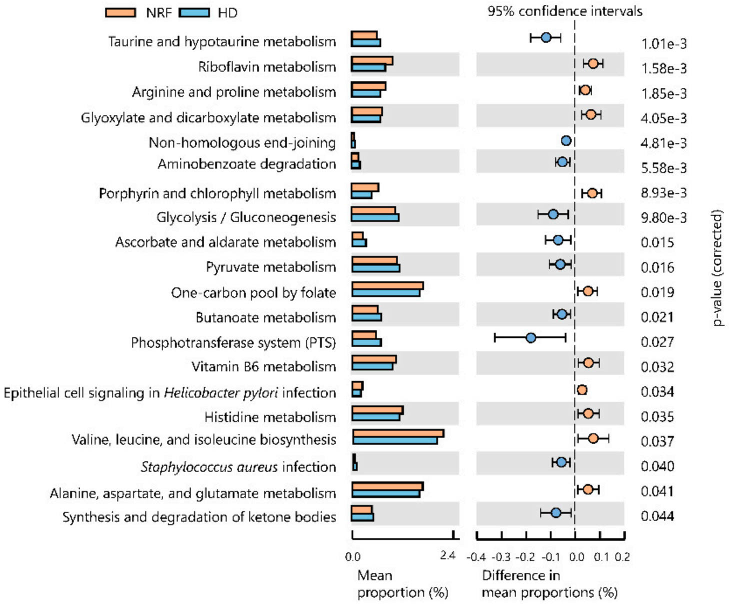

Supplementary figure S3B

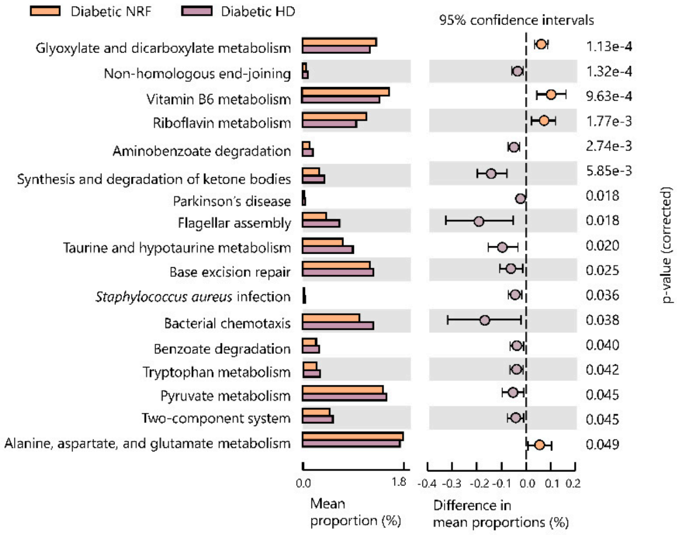

Supplementary figure S3C

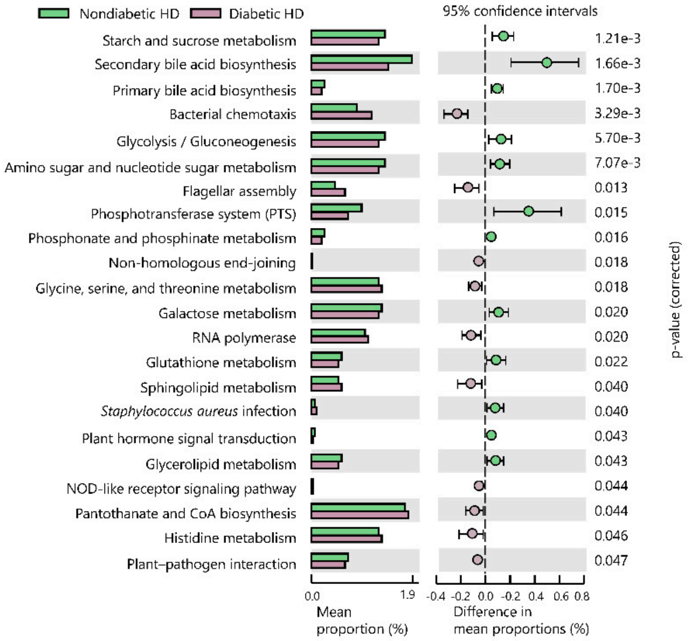

Supplementary Figure S3. Functional changes in the gut microbiome in each group. Characteristics of microbial function were compared in patients with NRF and those undergoing HD (Panel A), in diabetic patients with NRF and those undergoing HD (Panel B), and in patients undergoing HD with and without type 2 diabetes (Panel C).

Supplementary figure S4

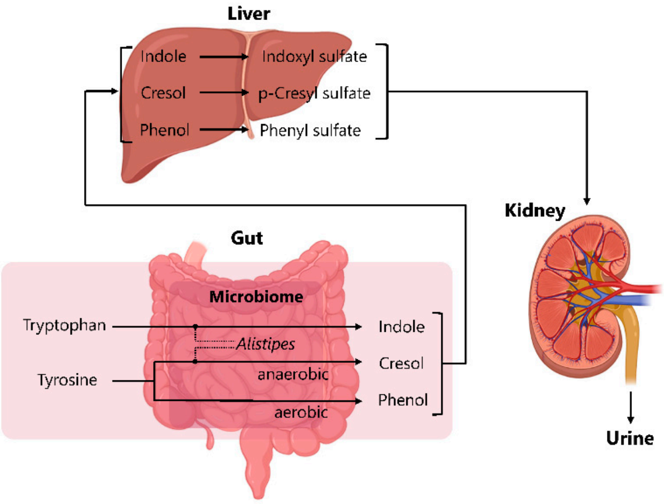

Supplementary Figure S4. Schematic illustration depicting the pathway of uremic toxin production. Indoxyl sulfate is produced from indole, which is derived from tryptophan. p-Cresyl sulfate and phenyl sulfate are produced from cresol and phenol, respectively, both of which are derived from tyrosine.
